# Real-world evidence in metabolic dysfunction-associated steatotic liver disease (MASLD): insights, challenges, and future directions

**DOI:** 10.1016/j.lanepe.2025.101557

**Published:** 2025-12-13

**Authors:** Huei-Tyng Huang, Michael Hewitt, Wenhao Li, William Alazawi

**Affiliations:** Barts Liver Centre and Barts Metabolism Network, Blizard Institute, Queen Mary University of London, 4 Newark St, London, E1 2AT, United Kingdom

**Keywords:** Real-world data, Non-communicable diseases, Epidemiology, Multimorbidity, Drug development

## Abstract

Real-world evidence (RWE), derived from real-world data, offers key insights into metabolic dysfunction-associated steatotic liver disease (MASLD). RWE complements traditional randomised controlled trials by capturing large-scale, diverse patient populations and long-term outcomes. This review interrogates the role of RWE in understanding MASLD epidemiology, natural history, hepatic and extra-hepatic endpoints, including its co-morbid association with cardiovascular and chronic kidney disease, and its use in pharmacovigilance and precision medicine. RWE highlights large regional variations and is increasingly leveraged to advance drug development through target discovery and patient stratification. However, challenges such as data quality and confounding factors persist. In this review, key methodological gaps and future research priorities are identified and highlighted. The potential of artificial intelligence with multi-modal data linkage is considerable but requires rigorous methodologies and collaboration to fully realise the potential of RWE in MASLD research.

## Graphical abstract

**Figure:**
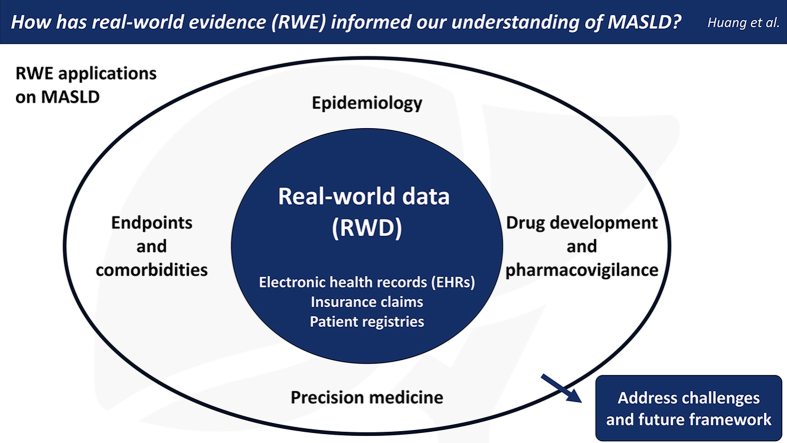

## Introduction

Real-world evidence (RWE) refers to clinical insights derived from the analysis of real-world data (RWD) — information about patients’ health status and the delivery of healthcare that is routinely collected from various sources. Unlike data collected from randomised controlled trials (RCTs), which are conducted in strictly controlled environments, RWD is gathered from everyday clinical settings such as electronic health records (EHRs), insurance claims and patient registries.^1^ As information technology within healthcare rapidly evolves and access to RWD improves, RWE is expected to play an increasingly vital role in enhancing the efficiency of clinical research and informing clinical practice.

**Key messages**

- Real-world evidence (RWE) has advanced understanding of metabolic dysfunction-associated steatotic liver disease (MASLD) epidemiology and natural history and established links with extra-hepatic disease.
- RWE can complement therapeutic randomised controlled trials by capturing long-term outcomes across diverse populations; therefore, supporting pharmacovigilance and potentially drug repurposing studies.
- Advanced analytics, including artificial intelligence and natural language processing, can leverage RWE for improved MASLD phenotyping.
- A dedicated framework to guide RWE gathering in MASLD is now needed.

**Search strategy and selection criteria**

A comprehensive literature search was initially performed using PubMed for articles published up to June 30, 2024, with the following terms: (“Non-alcoholic Fatty Liver Disease” OR “Non-alcoholic Steatohepatitis” OR “NAFLD” OR “NASH” OR “Metabolic dysfunction-associated steatotic liver disease” OR “Metabolic dysfunction-associated steatohepatitis” OR “MASLD” OR “MASH”). This search was systematically updated in August 2025 to ensure the inclusion of the most recent publications. Only English-language studies were included, and the final reference lists were generated according to the scope and relevance of this review.

RWE has several advantages. It can complement findings from RCTs, which, while considered the gold standard for clinical research, often involve narrowly defined populations under tightly controlled conditions and as such, may not fully represent the diverse populations and complex healthcare environments in everyday practice. RCTs typically use strict inclusion and exclusion criteria, whereas RWE potentially encompasses a broader range of demographics, comorbidities, and interventions, allowing the findings to be more applicable to the general population. RWE can also provide longitudinal data over extended periods. For instance, after registration in a national healthcare system, patients can be followed up indefinitely, allowing researchers to track changes through annual routine blood tests and identify long-term benefits or risks associated with specific data measurements. Additionally, RWE studies are often more time- and cost-efficient than RCTs. For example, during the COVID-19 pandemic, RWE played a key role in providing critical and almost contemporaneous data on prevalence, incidence, and transmission rates, as well as in assessing the effectiveness of vaccination programmes.^2^, ^3^, ^4^ These advantages make RWE increasingly attractive to researchers, regulators and policymakers.

However, despite its benefits, RWE also presents challenges. Concerns persist regarding the quality of the RWD used for RWE.^5^, ^6^, ^7^ RWD can be prone to bias, missing information, and heterogeneity in data collection methods across different sources. For example, data generated from EHRs in general practice may differ greatly from those recorded in a health insurance reimbursement system, which may different priorities in the details coded.^8^ This variability has the potential to compromise the validity of findings and limit causal inference, particularly as real-world studies lack randomisation.^9^ Moreover, RWD may lack key endpoints for analysis as patient registries and databases are not always designed with research in mind.

RWD have contributed greatly to our understanding of Metabolic dysfunction-Associated Steatotic Liver Disease (MASLD) and have the potential to provide further insights as therapies and data analytics evolve. Formerly referred to as non-alcoholic fatty liver disease (NAFLD), MASLD is a chronic liver disease characterised by the accumulation of fat in the liver (hepatic steatosis) in the context of metabolic risk factors.^10^ MASLD encompasses a spectrum that includes simple steatosis, metabolic dysfunction-associated steatohepatitis (MASH), fibrosis and ultimately cirrhosis. The updated nomenclature has been adopted to both reduce stigma and to more accurately reflect the central role of metabolic dysfunction — such as obesity, insulin resistance and dyslipidaemia — in disease pathogenesis and allows a positive diagnosis,^11^ rather than a diagnosis of exclusion. This review uses the MASLD terminology and although the majority of evidence cited is derived from cohorts defined under the old nomenclature, there is high concordance (all >94% from multiple studies)^12^, ^13^, ^14^ reported between these definitions, with international consensus also supporting NAFLD coding as the most practical proxy for MASLD in EHRs.^15^ MASLD diagnoses have relied on recorded alcohol intake which underestimates true consumption in both clinical and research settings, introducing potential selection bias. The growing recognition that alcohol-related liver disease and MASLD co-exist within the spectrum of steatotic liver disease (SLD) has also not been reflected in historic studies which may limit the generalisability of some findings as the field moves to embrace the broader SLD concept.

## Aim

In recent years, there has been a rapid expansion in the availability of RWD and advanced analytical methods, such as artificial intelligence (AI) and natural language processing (NLP), which offer new opportunities and challenges for studying MASLD at scale. This is particularly relevant for real-world efficacy and pharmacovigilance as resmetirom^16^ and semaglutide^17^ — and soon other drugs — will be used specifically for MASH and fibrosis. A synthesis of how RWE has been used to date in NAFLD provides a necessary foundation for guiding future MASLD research. This review evaluates how RWE has informed our understanding of MASLD epidemiology, disease endpoints (both hepatic and extrahepatic) as well as its growing utility within pharmacovigilance and describes novel emerging applications.

## Epidemiology

The global prevalence of MASLD is approximately 32% (95% confidence interval (CI): 30%–35%) based on systematic review of 72 studies.^18^ The estimates from these individual studies vary significantly across regions due to differences in data sources and study designs. Most of the evidence derives from cohort studies which often include high-risk individuals from specialist centres and may thus overestimate the prevalence in the general population. In the absence of a commonly-recorded biomarker of disease, detection of MASLD within RWD generally relies upon diagnostic coding, which itself is variable in accuracy. Inconsistent or incomplete use of diagnostic codes and variable clinician awareness may result in substantial under-diagnosis and under-recording of MASLD in RWD.^19^^,^^20^ Efforts to estimate MASLD prevalence using unselected RWD have also produced variable results that differ from the expected values. The estimated prevalence of MASLD in Australia in 2020 was 22.2% (95% CI: 19.0%–25.2%) using the Australia Bureau of Statistics national database.^21^ In Japan, the estimated prevalence was 9.2%, according to a health insurance claims database from the National Medical Center.^22^ In European primary care databases, prevalence of recorded diagnoses in routine health records increased from 0.6% (95% CI: 0.4%–0.8%) in 2007 to 1.9% (95% CI: 0.9%–2.8%) in 2014: substantially lower than estimates from cohort studies.^20^ These discrepancies likely reflect both genuine regional differences — such as in genetics, health behaviours and lifestyle including diet — and methodological limitations, particularly in diagnostic coding. In areas of the world where International Classification of Diseases (ICD) coding is used in EHRs, expert consensus is that MASLD should be coded for with K76.0 (MASLD) and K75.8 (metabolic dysfunction-associated steatohepatitis, MASH).^15^ However, there is evidence to suggest that reliance of ICD codes alone can miss over 40% of MASLD cases. Corey et al. demonstrated that including narrative data such as liver biopsy results can improve yield of MASLD diagnosis.^23^ Although the proportion of people undergoing liver biopsy is decreasing as non-invasive tools gain acceptance by health systems, this principle of utilising broader non-coded information held within the EHRs is apposite.

To overcome under-coding in health care records, some studies have calculated surrogate scores from available data to estimate the prevalence of MASLD within RWD. One such surrogate score is the “Fatty Liver Index” (FLI) — which combines triglycerides (TGs), body mass index (BMI), waist circumference, and gamma-glutamyltransferase (GGT) and has been shown to correlate with hepatic steatosis.^24^ However, the choice of FLI cutoff has a substantial impact on prevalence estimates: an FLI ≥60 yields prevalence rates between 5.5% and 9.8%, while a threshold of ≥30 can inflate rates up to 31.2%.^25^^,^^26^ Therefore, while potentially useful for correcting for under-coded data sets, there remains a risk of overdiagnosis when using surrogate markers, particularly in countries with a high social demographic index (SDI), where there is greater availability of advanced diagnostic tools and heightened awareness.^27^

**Fig. 1:**
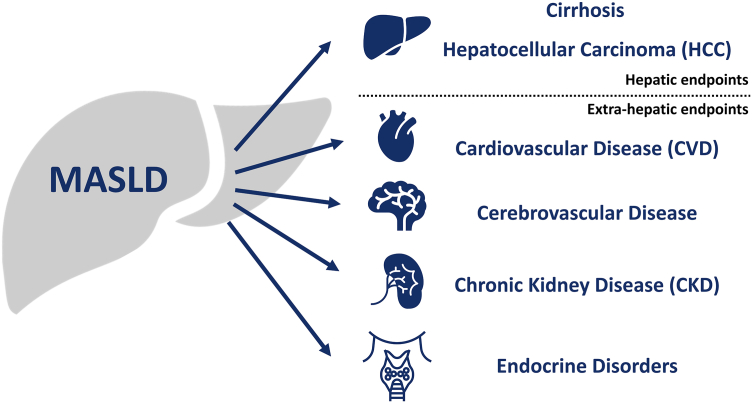
**The systemic impact of MASLD established by RWE**. RWE from large population-based studies has been crucial in defining the risk of hepatic and extra-hepatic complications associated with MASLD.

The following sections review the RWE linking MASLD to specific hepatic and extra-hepatic endpoints (Fig. 1). The studies cited are large, contemporary and illustrative of the variety of RWD sources currently in use albeit heterogeneous in methodology including how MASLD is diagnosed, completeness of confounder adjustment and demographics. The value of this evidence often comes not from a single, definitive strongest study, but from the consistency of the observed associations across diverse datasets.

## Hepatic endpoints

### Cirrhosis

RWD have been used to estimate the risk of progression from MASLD to more advanced stages of MASH, fibrosis and cirrhosis. In a combined analysis of four European RWD including over 18 million individuals, a diagnosis of MASLD/MASH was associated with a substantially increased risk of developing cirrhosis compared to matched controls, with a hazard ratio (HR) of 4.7 (95% CI: 2.4–9.2).^28^ Similarly, among 332,175 adults in the Taiwanese National Health Screening Program, a diagnosis of MASLD significantly increased the risk of cirrhosis compared to liver disease of non-steatotic aetiologies with a HR of 1.3 (95% CI: 1.2–1.4), even after adjusting for age, sex, smoking, and Fibrosis-4 Index.^29^ In this study, the presence or absence of steatosis was evaluated using ultrasonography, while cirrhosis was identified via ICD coding. The HR increased to 2.0 (95% CI: 1.8–2.3) when using individuals without any cardiometabolic risks as the reference group.^29^

### Hepatocellular carcinoma (HCC)

Individuals with MASLD/MASH are at a higher risk of developing HCC than those without.^30^ Data from the Swedish National Patient Register demonstrated that MASLD patients had markedly elevated risks of liver-related (HR: 26.9, 95% CI: 19.4–37.3) and HCC-specific mortality (HR: 35.0, 95% CI: 17.0–72.1).^31^ The Swedish Cancer Register also reported that MASLD has now become the most common aetiology of chronic liver disease in those who go on to develop HCC, with 22% of HCC diagnoses arising in individuals with a pre-existing diagnosis of MASLD.^32^

The Geneva Cancer Registry, which covers the entire population in the canton of Geneva, Switzerland (382,543 and 482,545 individuals in 1990 and 2014, respectively), reported an increase in proportion of MASLD-HCC, particularly in women (0%–29%) compared to men (2%–12%), specifically between the periods of 1990 to 1994 and 2010 to 2014.^33^ In a European RWE of more than 18 million individuals, the risks of developing HCC were 3.5 times higher (95% CI: 1.7–7.2) in those coded with MASLD/MASH compared to non-MASLD controls matched with age ± 5 years, sex, practice site and the practice visit records within ±6 months of the pseudo-index date.^28^ In South Korea, analysis of over six million individuals aged 40–79 years who underwent national health checks (2009–2010) showed that MASLD was associated with a 1.7-fold increased risk of HCC (95% CI: 1.6–1.7) compared to non-MASLD controls.^34^

US-based datasets support these findings. In the MarketScan claims database, MASLD was identified as the most common underlying liver disease in 59% of 4406 HCC cases.^35^ Similarly, in the US cancer registry database Surveillance, Epidemiology, and End Results (SEER), MASLD was the aetiology of underlying liver disease in 14.1% of 4929 HCC cases, with a 9% annual increase from 2004 to 2009.^36^

## Extra-hepatic endpoints

### Cardiovascular disease

RWE has consistently demonstrated that MASLD and MASH are risk factors for cardiovascular disease (CVD), including myocardial infarction, heart failure, and cardiovascular mortality. A European RWD of over 18 million individuals from four countries showed that coded MASLD was associated with a higher incidence of acute myocardial infarction (AMI), with an HR of 1.2 (95% CI: 1.1–1.3) after adjusting for age and smoking.^37^ Studies utilising the South Korean national database demonstrated HRs ranging from 1.2 to 1.7 with slightly different populations, follow-ups, and adjustments of confounders such as age, sex, and metabolic syndrome (MetS).^25^^,^^26^^,^^38^^,^^39^

MASLD significantly increased the risk of CVD regardless of stage in the Korean National Health Information Database, using FLI as a surrogate measure for severity of steatosis. Altering the cutoff of FLI ≥30 (HR: 1.2, 95% CI: 1.2–1.3) or ≥60 (HR: 1.4, 95% CI: 1.4–1.5) did not significantly impact the risk of CVD.^25^ These findings were corroborated in a national database of 6.8 million individuals from Japan, where the HR of developing CVD was 2.6 (95% CI: 2.4–2.8) after adjusting for age, sex, and smoking status; and 2.3 (95% CI: 2.2–2.5) for those who were overweight or obese, type 2 diabetic or were otherwise classified as having a MetS.^22^

Smaller, registry-based datasets also support this association. Analysis of the Swedish National Patient Register found that 10,422 patients with MASLD had a significantly higher incidence of major CVD, including ischaemic heart disease (HR: 1.6; 95% CI: 1.5–1.8), congestive cardiac failure (HR: 1.8; 95% CI: 1.6–1.9), and overall cardiovascular mortality (HR: 1.4; 95% CI: 1.3–1.5), when followed up over a median of 13.6 years.^40^

Importantly, CVD is also the leading cause of death in individuals with MASLD who do not have advanced fibrosis. Among 13,009 people with coded MASLD diagnoses in the Swedish National Causes of Death register, the estimated 15-year cumulative incidence of death due to CVD was 7.2% (95% CI: 6.4%–8.0%), accounting for around one-third of all deaths (all-cause mortality: 26.9%, 95% CI: 25.5%–28.4%) and surpassing other endpoints that we discuss in this review, including HCC (0.8%, 95% CI: 0.5%–1.1%).^31^

### Cerebrovascular disease

Across recent RWE, MASLD has also been repeatedly reported as an important risk factor for cerebrovascular disease and in particular, ischaemic cerebrovascular accidents (CVA). In a study analysing multiple large European RWD involving over 21 million individuals, MASLD was associated with a higher risk of stroke, with a HR of 1.2 (95% CI: 1.1–1.2) after adjusting for age and smoking.^37^ Four studies from South Korea, with populations ranging from 3 to 9 million, reported HRs for cerebrovascular events between 1.0 and 1.5, when considering different confounders, such as age, sex, household incomes, and exercise frequency.^25^^,^^26^^,^^38^^,^^39^

RWD from Japan further support this association. MASLD significantly increased the risk of cerebral infarction, with HRs of 1.3 (95% CI: 1.0–1.8) and 1.3 (95% CI: 1.0–1.7) after adjustment for other metabolic risk factors.^22^ Swedish national registry data showed an increased CVA risk among individuals with MASLD, reporting an HR of 1.6 (95% CI: 1.5–1.7) compared to matched controls.^40^

### Chronic kidney disease (CKD)

The association of MASLD with CKD is supported by evidence from large-scale RWE studies supporting this association. A real-world study involving 92,225 individuals with a coded diagnosis of MASLD matched 1:1 with non-MASLD individuals using the IQVIA Disease Analyzer Database suggested that individuals with MASLD had an increased risk of CKD with a HR of 1.9 (95% CI: 1.8–2.0) after adjusting for prescriptions of insulin and drugs affecting the kidneys such as angiotensin II receptor blockers and calcium channel blockers. They also demonstrated an 8% increase in CKD diagnoses (19.1% vs 11.1%) after 10 years of follow-up.^41^

Similarly, an analysis of over one million individuals from the US Truven Health MarketScan database revealed that MASLD patients had a 41% higher risk of developing advanced CKD (95% CI of HR: 1.4–1.5), with incidence rates of 8.2 and 5.5 per 1000 person-years for MASLD and non-MASLD individuals, respectively.^42^

Evidence from the National Health Service (NHS) iLFT (intelligent Liver Function Testing) database in Scotland, involving 2422 patients, further underscores this risk. The presence of CKD was associated with an increased mortality in individuals with MASLD particularly in those with fibrosis (determined by non-invasive tests), with adjusted HRs of 2.3 (95% CI: 1.5–3.6) for MASLD with fibrosis but no CKD, and 5.1 (95% CI: 3.1–8.4) for those with MASLD with fibrosis and CKD.^43^

### Endocrine disorders

While the majority of MASLD is diagnosed in individuals with pre-existing type 2 diabetes mellitus (T2DM), a diagnosis of MASLD in people without T2DM has been shown to be an independent risk factor for developing insulin resistance and diabetes. The prevalence of MASLD among T2DM patients ranges from 45% to 75% in hospital-based studies and 30% to 70% in population-based studies.^44^ Sakai et al. identified 2682 MASLD individuals from a Japanese health screening programme with 15,039 unselected participants, and 234 (8.7%) had developed T2DM at the end of data collection.^12^ A Brazilian multicentre study with 7073 non-diabetic individuals, including 2298 image-diagnosed MASLD, showed MASLD had a 1.9-fold (95% CI: 1.7–2.1) higher risk of developing T2DM when adjusting for 10 potential confounders, including smoking, alcohol intake, age and sex.^45^

While less well defined, MASLD is associated with non-diabetic endocrinopathies. Thyroid-stimulating hormone (TSH), free triiodothyronine (T3), and free thyroxine (T4) have all been reported to associate with fibrosis stage.^46^ Existing evidence on endocrine disorders after MASLD is mostly derived from small cohort studies. The Swedish nationwide histopathology cohort ESPRESSO, comprising 12,172 individuals with MASLD and 56,831 matched controls, revealed that those with hypothyroidism had a 1.7-fold increased odds of MASLD (95% CI: 1.4–2.1).^47^ A meta-analysis showed that primary hypothyroidism is associated with an increased risk of MASLD (OR: 1.4, 95% CI: 1.2–1.7; 24 studies), MASH and advanced fibrosis (OR: 2.8, 95% CI: 2.1–3.9; 5 studies).^48^ Sex hormone metabolism is also influenced by pathological mechanisms shared with MASLD, including insulin resistance, obesity, and dyslipidaemia. In men, lower testosterone levels correlated with a higher risk of MASLD^49^^,^^50^; in women, MASLD exacerbated insulin resistance and metabolic dysregulation in polycystic ovary syndrome (PCOS).^51^

### Clinical review

Taken together, RWE complements findings from cohort studies that people living with MASLD are at increased risk of significant co-morbid cardio- and cerebrovascular disease and CKD. Many shared risk factors, such as hypertension, dyslipidaemia and chronic low-grade inflammation are likely contributors and some studies suggest an additional effect of MASLD independent of these,^44^^,^^52^ highlighting potential patient benefits of integrated care models for managing metabolic comorbidities in this population.

## Supporting drug development

The advent of approved drugs for MASLD in the USA and Europe and the ongoing clinical trials landscape is transforming the clinical care of patients with MASLD. The drug development process remains lengthy and expensive. However, RWE offers several opportunities to improve efficiency and increase the likelihood of success throughout the drug development lifecycle.^16^ One of the most powerful applications of RWE is in drug repurposing. Many drugs currently approved for metabolic co-morbidities, such as T2DM, may also prove effective in treating MASH, and RWE can give insights into the likelihood of a beneficial effect on MASLD/MASH and its associated endpoints and also in which specific populations.^53^ This approach supports drug repurposing efforts by identifying benefits in MASLD patients without requiring dedicated trials. This aligns with the American Diabetes Association's 2025 Standards of Care, which suggests that optimising pharmacologic treatment in patients with type 2 diabetes may also benefit MASLD management.^54^ Furthermore, RWD can be used to optimise clinical trial design by identifying patient subgroups with specific characteristics who are at higher risk of progression, thereby enriching recruitment with individuals most likely to benefit from treatment. TARGET-NASH is a real-world study of glucagon-like peptide-1 receptor agonists (GLP-1 RA) in 4219 individuals with MASLD or MASH. Compared to the 375 individuals prescribed a GLP-1 RA for diabetes or obesity indications, those not on GLP-1 RA treatment had over twice the risk of all-cause mortality (adjusted HR: 2.3; 95% CI: 1.4–3.6) and a 1.7 (95% CI: 1.2–2.5) times greater hazard of progression to decompensated cirrhosis.^55^ RWE can also be used to construct external or synthetic control arms for single-arm trials, providing comparator groups that reflect standard-of-care outcomes in similar populations.^56^

### Pharmacovigilance

Pharmacovigilance, defined by the World Health Organization (WHO) as “the science and activities relating to the detection, assessment, understanding and prevention of adverse effects or any other medicine/vaccine related problem” is a crucial element of safe and effective clinical care. The availability of more comprehensive RWE has driven a shift in pharmacovigilance that now incorporates RWE for post-regulatory approval and marketing instead of relying solely on clinical trials^57^ which may not capture rare or long-term adverse drug reactions.^57^^,^^58^ RWE captures broader, more heterogeneous patient populations and longer-term outcomes, thereby enhancing ongoing safety and efficacy monitoring and has been used to positive effect in other metabolic diseases.^59^

This is particularly important for MASLD and MASH, where trials are still of relatively short duration and there has been insufficient use of resmetirom in individual centres to provide cohort data. Hence there is a need to federate real-world datasets and generate RWE. Underreporting of adverse events (AEs) and adverse drug reactions (ADRs) — particularly liver-related ADRs — is a major limitation in pharmacovigilance.^58^ Contributory factors to this include awareness, uncertainty about what to report, time constraints and challenges in determining causality.^60^ These are human factors which RWD have the potential to overcome with consistent guidance to ensure their appropriate use in regulatory decisions.^61^

Outside the EHR, a wealth of health-related data are generated using health technology and near patient personal devices. Involving patients more directly in safety monitoring through digital tools such as apps for reporting side-effects, clinical and patient-related outcomes can improve ADR detection and increase the volume of RWE.^57^ However, patient-generated content can be subjective and cannot be verified by the researchers. Comments may be biased towards negative experiences and the context in which associations are made can be difficult to ascertain, creating an imbalance in ‘signal-to-noise’.

### Health economics

Accurate health economic assessments are essential for informing policy decisions and ensuring appropriate allocation of resources. It relies heavily on precise estimates of disease burden and transitions between disease stages. According to the Global Burden of Diseases (GBD) database for the general population^62^ MASLD incidence peaked in countries with a moderate SDI level, and with an average of 0.5 years lived with disability per affected individual (95% CI: 0.3–0.8). Although the GBD database itself is not primary RWD, it is derived from and based on multiple RWD sources spanning more than 200 countries. This aggregation of diverse datasets to produce robust, population-level estimates exemplifies how RWE can inform policymakers and support the case for allocating resources to hepatology services.

## RWD and the future

### Precision medicine

Precision medicine tailors diagnosis or treatment to the individual characteristics of a patient, incorporating genetic and phenotypic factors to optimise therapeutic efficacy and minimise adverse effects. In the context of MASLD, precision medicine aims to address the heterogeneity by enabling complex phenotyping, individualised risk prediction, and the development of targeted therapeutic strategies. As our understanding of MASLD pathogenesis grows, it is increasingly clear that MASLD represents a spectrum of distinct subtypes rather than a single disease entity. Identifying a patient's predominant subtype can support a precision medicine approach, informing personalised strategies for surveillance (e.g. cardiovascular monitoring vs frequent liver fibrosis assessment) and the selection of targeted therapeutic agents.^63^ The integration of multi-omic (including genetic) data with clinical information — often derived from RWD — offers significant potential for patient stratification and therapeutic targeting.

Genetic variants such as *PNPLA3* and *TM6SF2* have been implicated in the pathogenesis of MASLD^64^ and polygenic risk scores (PRS) that aggregate the effects of multiple variants, are associated with disease severity and outcomes.^65^ Although evidence for PRS generally comes from cohort studies, genetic variants can be integrated with EHR-linked data to contribute to risk stratification, in projects such as Genes and Health, FinnGen and UK Biobank (UKBB).^66^, ^67^, ^68^ For example, a study pooling multiple databases with EHR-diagnosis found that a set of genes including *PNPLA3* is associated with more severe steatohepatitis and fibrosis, and individuals in the top 10% of PRS have a 2.5-fold and 2.9-fold increased odds of cirrhosis (95% CI: 2.0–3.2), and HCC (95% CI: 1.8–4.8) compared to those with PRS in the bottom decile.^69^ However, it is worth noting that such a study is not a strictly unselected RWE, as the largest proportion of samples (65% of imaging and 51% of coded diagnosis, respectively) came from UK Biobank, a participant-recruited population who have tended to suffer healthy volunteer bias.

### Artificial intelligence

AI and machine learning (ML) have been employed to analyse complex RWD, offering powerful new ways to diagnose, phenotype, and predict outcomes in patients with MASLD. Several key applications have emerged, which are summarised in Table 1.

**Table 1:** Examples of artificial intelligence applications in MASLD research.

AI techniques	Author	Data Source	Key aim	Main findings	Reference
Clustering	Raverdy et al. (2024); Jamialahmadi et al. (2024)	Multiple independent cohorts	To identify distinct MASLD subtypes	Both studies identified two similar subtypes: a ‘cardiometabolic’ subtype and a ‘liver-specific’ subtype with low CVD risk.	^70^^,^^71^
Machine learning	McTeer et al. (2024)	European NAFLD Registry	To predict histological characteristics from routine clinical variables	High accuracy in predicting MASLD outcomes: - clinically significant fibrosis (AUC: 0.85) - advanced fibrosis (AUC: 0.96) - cirrhosis (AUC: 0.99)	^72^
Multi-modal machine learning models	Kendall et al. (2023)	Scottish cohort SteatoSITE (histopathology + EHR)	To predict hepatic decompensation events using a gene score	A 15-gene risk score accurately predicted the 1-, 3-, and 5-year risks of decompensation (AUCs: 0.86, 0.81, 0.83).	^73^
Convolutional neural network (CNN)	Han et al. (2020)	Cohort with radiofrequency ultrasound data	To diagnose MASLD and quantify liver fat from ultrasound images	CNN achieved 96% (95% CI: 90%–99%) accuracy in diagnosing MASLD and estimating hepatic fat fraction compared to the MRI-PDFF reference standard.	^74^
Natural language processing (NLP)	John et al. (2025)	US Veterans Health Administration (VHA) Corporate Data Warehouse	To identify under-coded MASLD cases from clinical texts	Identified 36.5% (N = 299,259) of veterans with MASLD, compared to only 2.8% (N = 23,218) using ICD codes, an over 10-fold increase.	^75^

RWE from large population-based studies has been crucial in defining the risk of hepatic and extra-hepatic complications associated with MASLD.

Abbreviations: CVD, cardiovascular disease; AUC, area under curve; EHR, electronic health records; MRI-PDFF, magnetic resonance imaging-derived proton density fat fraction; ICD, International Classification of Diseases (ICD) coding.

AI applied to clustering analyses of multi-omic datasets, has helped identify some of the distinct risk profiles within MASLD subtypes^70^^,^^71^ and ML models trained on routine clinical or transcriptomic data have shown high accuracy in predicting histological severity and the progression to clinical endpoints.^72^^,^^73^ AI is increasingly transforming the diagnostic landscape of MASLD. Convolutional neural networks (CNNs), a class of AI that is capable of pattern recognition in imaging data, have been used to diagnose MASLD and quantify hepatic steatosis from ultrasound data with accuracy comparable to that of MRI-derived proton density fat fraction (PDFF).^74^ Perhaps most impactful is the advent of NLP, which, when applied to unstructured clinical text within EHRs, has substantially improved case identification.^75^ This development suggests that an algorithm combining rule-based NLP with cardiometabolic risk factors and alcohol use from EHRs could outperform the standard medical codes currently used in EHRs in identifying MASLD cases. In addition, given the predicted global prevalence of MASLD is approximately 30%, as evidenced by both the above NLP results and the pooled global meta-analysis,^76^ it follows that current coded-EHR approaches may misclassify over 90% of MASLD cases as controls. NLP applications in MASLD include extracting relevant clinical information to identify under-coded or missed cases, the detection of sub-phenotypes and comorbidities from narrative diagnoses, and enhancing diagnostic and staging processes through ML.

### RWE research framework

RWE has the potential to support and complement the global research priorities agenda recently proposed for MASLD^77^ which includes the economic burden of the disease, models of care, and approaches to education and community engagement. (main challenges and corresponding research priorities for the field in Table 2). To achieve this, a dedicated framework to guide RWE gathering in MASLD is needed. Such a framework should be built with expert consensus that addresses such domains as data completeness and coding, data access and sharing and statistical and analytical methods. The framework should promote the value of effective data entry at the clinical encounter to patient-relevant discoveries. In the absence of a single, definitive diagnostic or prognostic tool, it is likely that MASLD RWD will require data integration from different stakeholders (laboratory, imaging, primary, secondary and social care) and so guidance on managing these data flows and analytical techniques is needed. Within the safeguards of data custodianship, data curation should be balanced with democratised data access for clinical and research purposes and should avoid complex, tiered or preferential data access processes.

**Table 2:** Summary of challenges and future research priorities for RWE in MASLD.

Domain	Current challenge	Future research priority
Data quality and coding	Substantial under-coding of MASLD in EHRs limits case identification.	Develop and validate advanced algorithms (e.g. NLP) to improve case ascertainment from structured and unstructured EHRs.
Disease spectrum	Difficulty in distinguishing MASLD from other SLD (e.g. MetALD) due to underreported alcohol consumption in RWD.	Utilise multi-modal RWD to better phenotype patients across the SLD spectrum and study the natural history of MetALD.
Endpoint ascertainment	Lack of granular, research-grade endpoints (e.g. fibrosis progression) in most routine RWD sources.	Link EHRs with more specialised data sources (e.g. imaging archives, pathology reports, genomics) to create robust datasets for studying disease progression.
Methodology	High risk of bias and confounding inherent in non-randomised RWD, limiting causal inference.	Apply advanced statistical methods (e.g. target trial emulation) to RWD to more robustly estimate treatment effectiveness and comparative safety.
Pharmacovigilance	Underreporting of adverse drug reactions in routine care settings limits long-term safety monitoring for new MASLD therapies.	Integrate PROMs and data from digital health tools to create a more comprehensive safety surveillance system.

Abbreviations: EHRs, electronic health records; NLP, natural language processing; SLD, steatotic liver diseases; MetALD, metabolic dysfunction and alcohol-related liver disease; PROMs, patient-reported outcome measures.

## Conclusion

RWE have advanced our understanding of MASLD epidemiology, natural history and most recently, drug development in more representative patient populations than traditional RCTs permit. There are enormous opportunities to link with platform and digital science to enhance and deepen our analyses for patient benefit. However, significant challenges in data completeness and veracity persist. Addressing these issues requires standardised diagnostic frameworks and advanced data extraction and analytic techniques. Continued innovation, paired with practical implementation strategies, will be essential to translate real-world insights into meaningful improvements in patient care, policy, and health outcomes.

## Contributors

HTH: Conceptualisation, Methodology, Investigation, Writing — Original Draft, Review & Editing.

MH: Conceptualisation, Investigation, Writing — Review & Editing.

WL: Writing — Review & Editing. WA: Conceptualisation, Supervision, Writing — Review & Editing.

## Declaration of interests

WA has received grants to his institute from Gilead Sciences, GSK and MSD. He has received fees for consulting and honoraria for lectures from 89Bio, Akero, Echosens, Gilead Sciences, Goldman Sachs, GSK, Intercept, Inventiva, Janssen, Madrigal Pharmaceuticals and Novo Nordisk.
